# Survival of Patients with Hepatocellular Carcinoma (HCC) Treated by Percutaneous Radio-Frequency Ablation (RFA) Is Affected by Complete Radiological Response

**DOI:** 10.1371/journal.pone.0070016

**Published:** 2013-07-29

**Authors:** Giuseppe Cabibbo, Marcello Maida, Chiara Genco, Nicola Alessi, Marco Peralta, Giuseppe Butera, Massimo Galia, Giuseppe Brancatelli, Claudio Genova, Maurizio Raineri, Emanuele Orlando, Simona Attardo, Antonino Giarratano, Massimo Midiri, Vito Di Marco, Antonio Craxì, Calogero Cammà

**Affiliations:** 1 Dipartimento Biomedico di Medicina Interna e Specialistica, Sezione di Gastroenterologia, Università di Palermo, Palermo, Italia; 2 Dipartimento di Biopatologia e Biotecnologie Mediche e Forensi, Sezione di Radiologia, Università di Palermo, Palermo, Italia; 3 Dipartimento di Biopatologia e Biotecnologie Mediche e Forensi, Istituto di Anestesia e Rianimazioma, Università di Palermo, Palermo, Italia; University of Navarra School of Medicine and Center for Applied Medical Research (CIMA), Spain

## Abstract

**Background:**

Radio-frequency ablation (RFA) has been employed in the treatment of Barcelona Clinic Liver Cancer (BCLC) early stage hepatocellular carcinoma (HCC) as curative treatments.

**Aim:**

To assess the effectiveness and the safety of RFA in patients with early HCC and compensated cirrhosis.

**Methods:**

A cohort of 151 consecutive patients with early stage HCC (122 Child-Pugh class A and 29 class B patients) treated with RFA were enrolled. Clinical, laboratory and radiological follow-up data were collected from the time of first RFA.

A single lesion was observed in 113/151 (74.8%), two lesions in 32/151 (21.2%), and three lesions in 6/151 (4%) of patients.

**Results:**

The overall survival rates were 94%, 80%, 64%, 49%, and 41% at 12, 24, 36, 48 and 60 months, respectively. Complete response (CR) at 1 month (p<0.0001) and serum albumin levels (p = 0.0004) were the only variables indipendently linked to survival by multivariate Cox model. By multivariate analysis, tumor size (p = 0.01) is the only variable associated with an increased likehood of CR.

The proportion of major complications after treatment was 4%.

**Conclusions:**

RFA is safe and effective for managing HCC with cirrhosis, especially for patients with HCC ≤3 cm and higher baseline albumin levels. Complete response after RFA significantly increases survival.

## Introduction

The extensive application of surveillance programs for early detection of hepatocellular carcinoma (HCC) has increased the number of tumors detected within the Milan criteria [1] and potentially responsive to curative treatments, such as liver transplantation, resection and percutaneous or surgical ablation. [2], [3] Radiofrequency ablation (RFA) is currently recognized as an effective local treatment [2] in patients with Barcelona Clinic Liver Cancer (BCLC) early stage hepatocellular carcinoma (HCC) not elegible to surgical treatments. However, the 5-year survival rate of patients with HCC 5 cm after RFA is lower 50% because of the high risk of recurrence [4] and the influence of the severity of the underlying liver disease.

The aims of this prospective cohort study of BCLC early stage HCC patients treated with RFA as first-line treatment were:

1. to assess the effectiveness of RFA in HCC patients;
2. to select the optimal candidate for RFA by identification of predictors of overall survival.

## Materials and Methods

### Patients

From January 2000 to May 2011, 825 consecutive patients with cirrhosis and a new diagnosis of HCC were observed at our Liver Unit.

Cirrhosis was diagnosed by histological or clinical features and the liver function was evaluated according to Child-Pugh score. The diagnosis of HCC was performed by ultrasound guided biopsy or by multiphasic contrast-enhanced computed tomography or gadolinium-enhanced magnetic resonance.

All patients were evaluated according to European Association for the Study of the Liver criteria [5] up to 2005, and to American Association for the Study of Liver Diseases criteria [6] from January 2006. HCC staging and the choice of treatment were performed according to the BCLC algorithm. [7].

Extra-hepatic disease was assessed with multidetector multiphasic CT and chest radiography. Bone metastases were sought by scintigraphy if clinically suspected.

In the study period 151 were treated by RFA as first-line treatment. In all cases a multidisciplinary panel including a liver surgeon, an interventional radiologist, and hepatologists reviewed the patients’ files and imaging examinations. Inclusion criteria were: (a) tumor detectable by ultrasound (US) with an acceptable and safe path between the lesion and skin as shown on US, (b) patients who refused surgery or orthotopic liver trasplantation or with associated diseases contraindicating surgery; (c) a single HCC of 5 cm in diameter or smaller or as many as three HCCs each 3 cm in diameter or smaller; (d) cirrhosis classified as Child-Pugh class A or B; (e) absence of portal vein thrombosis or extrahepatic metastases; (f) platelet count >40,000/ mm3; prothrombin time ratio >40%. Exclusion criteria were tumors >5 cm, extrahepatic metastases, portal vein thrombosis, ascites, dilated bile ducts or cardiac arrhythmia. We used a commercial ultrasound (US) scanner (ESAOTE biomedica AU5) with a guide device. Before starting the procedure, all the patients were staged with spiral computed tomography (CT) scan, alfa-fetoprotein (AFP), and chest X-ray. The mean time elapsed between HCC diagnosis and the RFA procedures was 1 month.

The study was approved by the local ethics committee of the “Azienda Ospedaliera Universitaria Policlinico Paolo Giaccone” and a written informed consent was obtained from all patients.

### RF Ablation Procedures

RFA was performed under US guidance with a 150W generator (Model 1500 L; RITA Medical System, Mountain View, Calif.), connected to an expandable 15–14-gauge electrode with a 2.0-cm-long exposed tip (expandable by means of seven hooks). After administration of analgesia (50 to 60 mg of propofol and 0.05 to 0.1 mg of fentanyl) as well as local anesthesia (5 to 15 mL of 1% lidocaine) by an anesthesiologist, a RFA needle was first inserted into the tumor. The electrode was placed into the centre of the lesion maintaining the temperature of the needle tip at 100°C or more for 10–12 min. After ablation, the needle was retracted maintaining its tip hot in order to prevent by thermal coagulation seeding or haemorrhage along the electrode track. For patients with multiple tumors, all lesions were treated in one single session.

### Outcomes and Follow-up

The primary outcome was overall survival. Follow-up time was defined as the number of months from first RFA to orthotopic liver transplantation (OLT), last contact with the patient, or death.

Response rate magnitude was defined according to the European Association for the Study of the Liver (EASL) criteria. [5] These are addressed by recent guidelines. [3], [8] EASL response criteria are defined as follows:

- complete response (CR), defined as absence of any enhancing tissue;
- partial response (PR), defined as >50% decrease in enhancing tissue;
- and stable disease, defined as <50% decrease in enhancing tissue.

Progressive disease is defined by at least a 25% increase in enhancing tissue of the treated tumor. Treatment failure was defined as the presence of viable tumor at the end of therapy.

The follow-up protocol included clinical assessment by physical examination, ultrasound scan and biochemistry every 3 months, and by multiphasic CT scan every 6 months. In our study, RFA-related morbidity was defined as any complication within 2 weeks of each session of RFA. RFA-related mortality was defined as death from a complication within 2 weeks of each session of RFA.

### Statistical Evaluation

Data were collected by experienced medical personnel involved in the study using a common electronic database. The primary outcome weas assessed by intention-to-treat, while continuous variables were expressed as mean standard deviation. Categorical variables were analyzed as frequency and percentages. The Kaplan-Meier model was used to estimate survival. Differences in the survival rate were assessed by log-rank testing. Potential prognostic variables were evaluated as predictors of survival by Cox models. All variables at baseline included in Table 1, with the addition of treatment response, and number of treatments, during follow-up, were studied at univariate analyses. Variables with a p value of <0.10 at univariate analysis were included in the final multivariate model. To avoid the effect of co-linearity with albumin, bilirubin and International Normalised Ratio, Child-Pugh score was not included in the same multivariate model. Multiple logistic regression model was used to assess the relationship of complete radiological response with the demographic, laboratory, clinical, and tumor staging characteristics of patients. For all analyses, P≤0.05 was considered statistically significant. All p values were two-tailed, and all confidence intervals (CIs) were 95%. All multivariate analyses were done with PROC PHREG in SAS version 8.1 (SAS Institute, Inc, Cary, NC, USA).

**Table 1:** Demographic, Laboratory, Clinical and Tumor Staging Characteristics of 151 Patients with HCC in Compensated Cirrhosis Treated with RFA.

Characteristic	Child-Pugh A (n = 122)	Child-Pugh B (n = 29)
**Age – years**	67±7.5	67±5.4
**Male – no. (%)**	82 (67)	20 (69)
**Etiology of Cirrhosis-no. (%)**		
Anti-HCV positivity	108 (88.4)	25 (86.4)
HBsAg positivity	10 (8.2)	1 (3.4)
Alcohol abuse	3 (2.6)	1 (3.4)
NAFLD	1 (0.8)	2 (6.8)
**Hepatic Encephalopathy-no. (%)**		
None	122 (100)	29 (100)
**Ascites – no. (%)**		
None	122 (100)	29 (100)
**Albumin** – **g/dl**	3.7±0.5	3.2±0.4
**International Normalised Ratio**	1.10±0.12	1.2±1.5
**Total Bilirubin – mg/dl**	1.2±0.9	2.1±0.5
**Platelet × 10^3^/** **mmc**	105±58	72±32
**Creatinine-mg/dl**	0.8±0.3	0.9±0.3
**MELD Score**	9.0±1.9	9.0±2.1
**Oesophageal varices – no. (%)**		
None	53 (43.5)	5 (17.2)
F1	48 (39.3)	16 (55.2)
F2/F3	21 (17.2)	8 (27.6)
**Portal Vein Thrombosis – no. (%)**		
None	122 (100)	29 (100)
**AFP - ng/mL**	75±115	132±633
**Number of Nodules (%)**		
1	90 (73.3)	23 (79.3)
2	27 (22)	5 (17.2)
3	5 (4.7)	1 (3.5)
**Tumor size– no. (%)**		
3 cm	103 (84.4)	25 (86.2)
**>**3 cm	19 (15.6)	4 (13.8)

Note. Values are mean ± SD.

Abbreviations: IU, international units; AFP, alpha fetoprotein; HCV, hepatitis C virus; HBV, hepatitis B virus; NAFLD, Nonalcoholic fatty liver disease; MELD, Model for End-Stage Liver Disease.

## Results

### Patient Features at Baseline

The study population consisted of 151 patients with HCC secondary to cirrhosis of different etiologies treated by RFA. The demographic, clinical and tumor staging features of the 151 patients are given in Table 1. Chronic HCV infection was the dominant etiology, followed by chronic hepatitis B virus (HBV) infection.

At presentation 122 of the 151 patients (81%) were in the Child-Pugh A class. None of the 29 Child-Pugh B patients had ascites, jaundice, or hepatic encephalopathy.

A single lesion was observed in 74.8%, and two or three lesions in 25.2% of patients.

The tumor size was ≤3 cm 128/151 (84.8%) of the patients.

### Follow-up

The mean length of follow-up after RFA was 35.6±26.7 months (median 29.6 months, range 4–139 months). Six (4%) patients, lost during follow-up, were censored at the last visit. During follow-up, 71 patients died, while 80 (53%) were alive at the end of the study (Table 2).

**Table 2:** Follow-up of 151 Patients with HCC in Compensated Cirrhosis Treated with RFA.

Characteristics	Value
**Complete response-no. (%)**	118 (78)
after one cycle	109 (92)
after two cycles	9 (8)
**Recurrence after compete response-no. (%)**	80 (67.8)
local recurrence	46 (39)
distant recurrence	34 (28.8)
**Complications after RFA – no. (%)**	5 (4)
portal vein thrombosis	1 (0.8)
subcapsular hematoma	1 (0.8)
intrahepatic abscess	1 (0.8)
pleural effusion	1 (0.8)
seeding	1 (1.8)
**Other Treatments – no. (%)**	87 (57.6)
RFA	41 (27.1)
TACE	26 (17.2)
OLT	2 (1.3)
RFA+TACE	7 (4.6)
Sorafenib	11 (7.3)
**Extra-hepatic spread – no. (%)**	11 (7.3)
**Overall Survival, Median -mo (95% CI)**	47.6 (41–61)
Child-Pugh A (n = 122)	59.4 (48–72)
Child-Pugh B (n = 29)	26 (15–41)
**Recurrence-Free Survival, Median -mo (95% CI)**	17 (13–21)
**Death -no. (%)**	71 (47)

Abbreviations: TACE, Transarterial chemoembolization; OLT, orthotopic liver transplantation; PEI, percutaneous ethanol injection; RFA, radiofrequency thermal ablation; CI, confidence interval.

CR after RFA was achieved in 118 patients (78%), in 109 after one session, in 9 after two sessions (no significant differences in term of tumor staging characteristics were found in this subgroup of patients). Treatment failure was observed in 33/151 patients (22%), including 10 patients with 3 HCC nodules, 15 patients with HCC nodules larger than 4 cm and 8 patients with HCC nodules located in areas difficult to reach. During follow-up, 80 of 118 patients who achieved CR developed recurrences (local recurrences in 46 patients, while distant recurrences in 34 patients). Median time to recurrences was 17 months (range 13–21).

**Figure 1:**
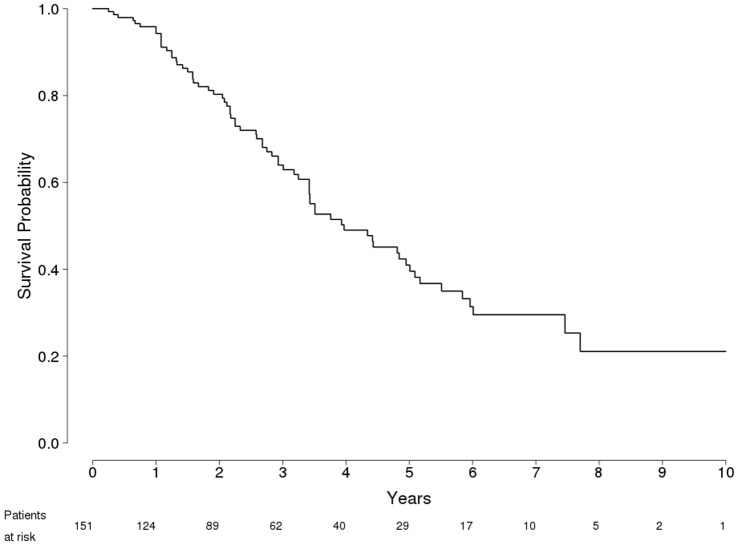
Probability of Overall Survival in 151 Patients with HCC in compensated Cirrhosis Treated with RFA.

Median overall survival after RFA was 47.6 months (95% confidence interval [CI] 41–61.1) for the entire group. Median survival was longer in patients who achieved CR after RFA (59.4 mo, 95%CI 50.8–68.0) than among patients who failed treatment (26 mo, 95%CI 19.4–32.5). The overall survival rates (Figure 1) were 94%, 80%, 64%, 49%, and 41% at 12, 24, 36, 48 and 60 months, respectively.

**Figure 2:**
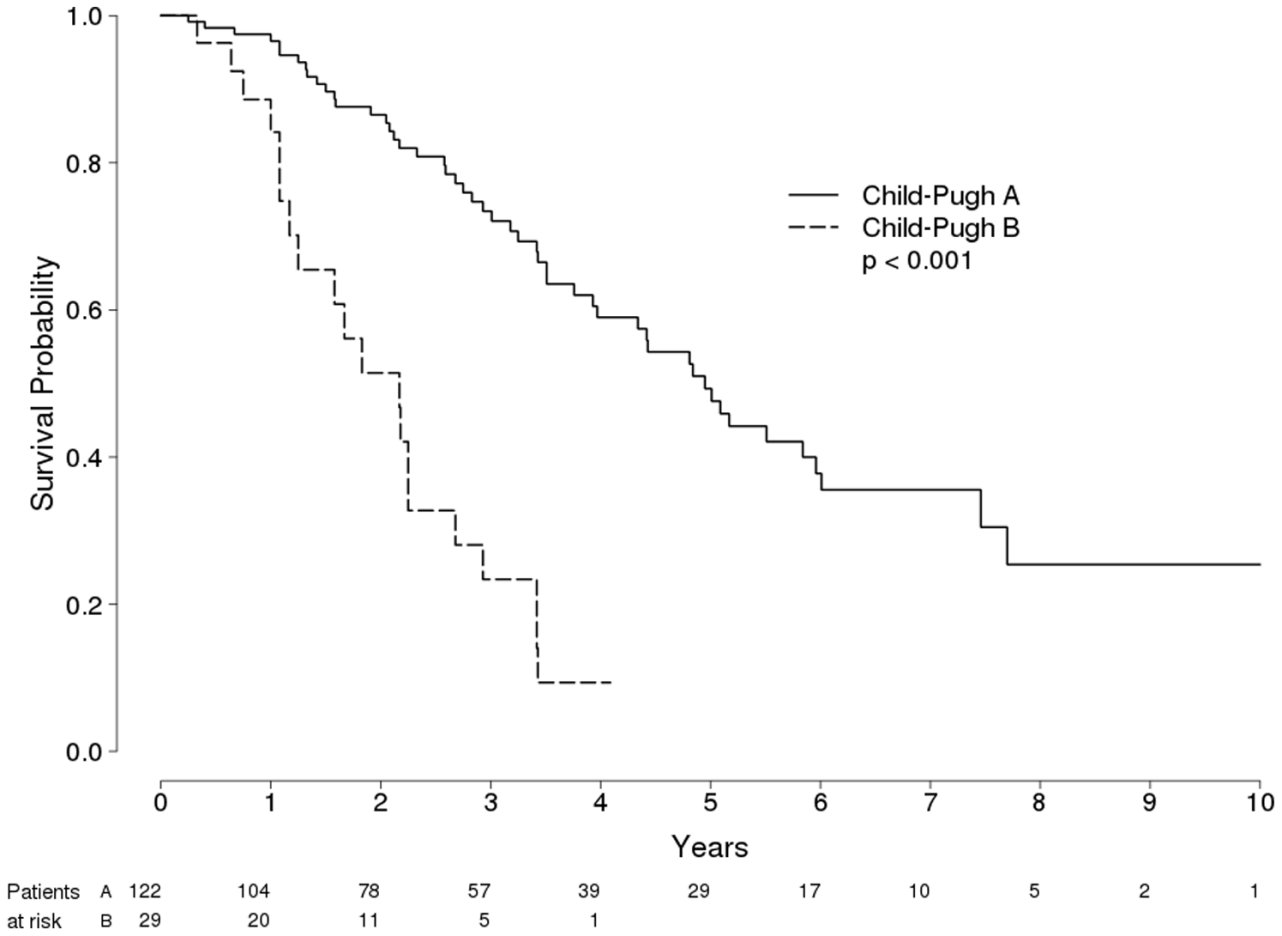
Probability of Overall Survival in 151 Patients with HCC in compensated Cirrhosis Treated with RFA, according to Child-Pugh classes.

**Figure 3:**
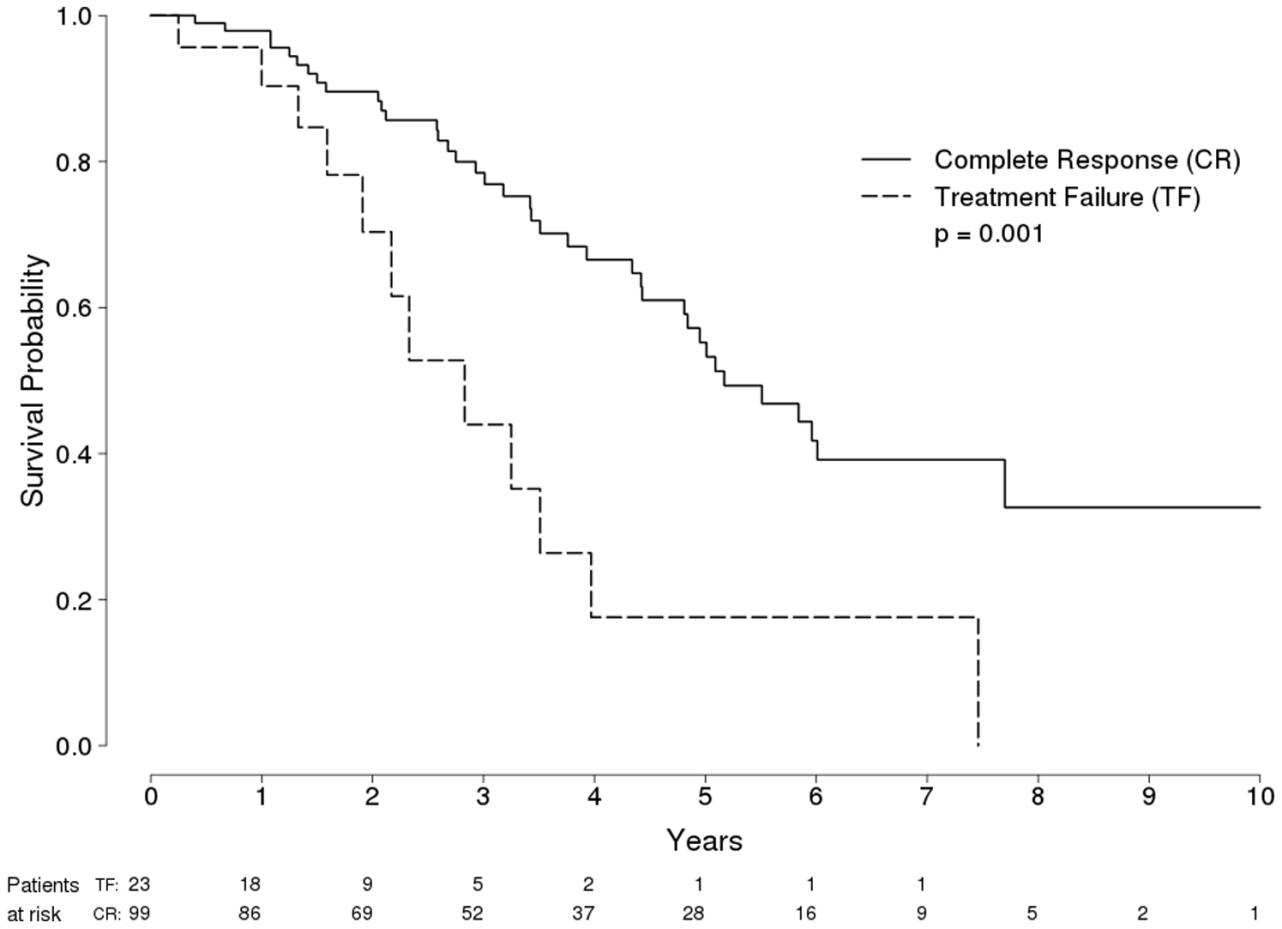
Probability of Overall Survival in 122 Child-Pugh A Patients with HCC Treated with RFA, according to radiological response (Complete response [solid line] vs treatment failure [dashed line]).

**Figure 4:**
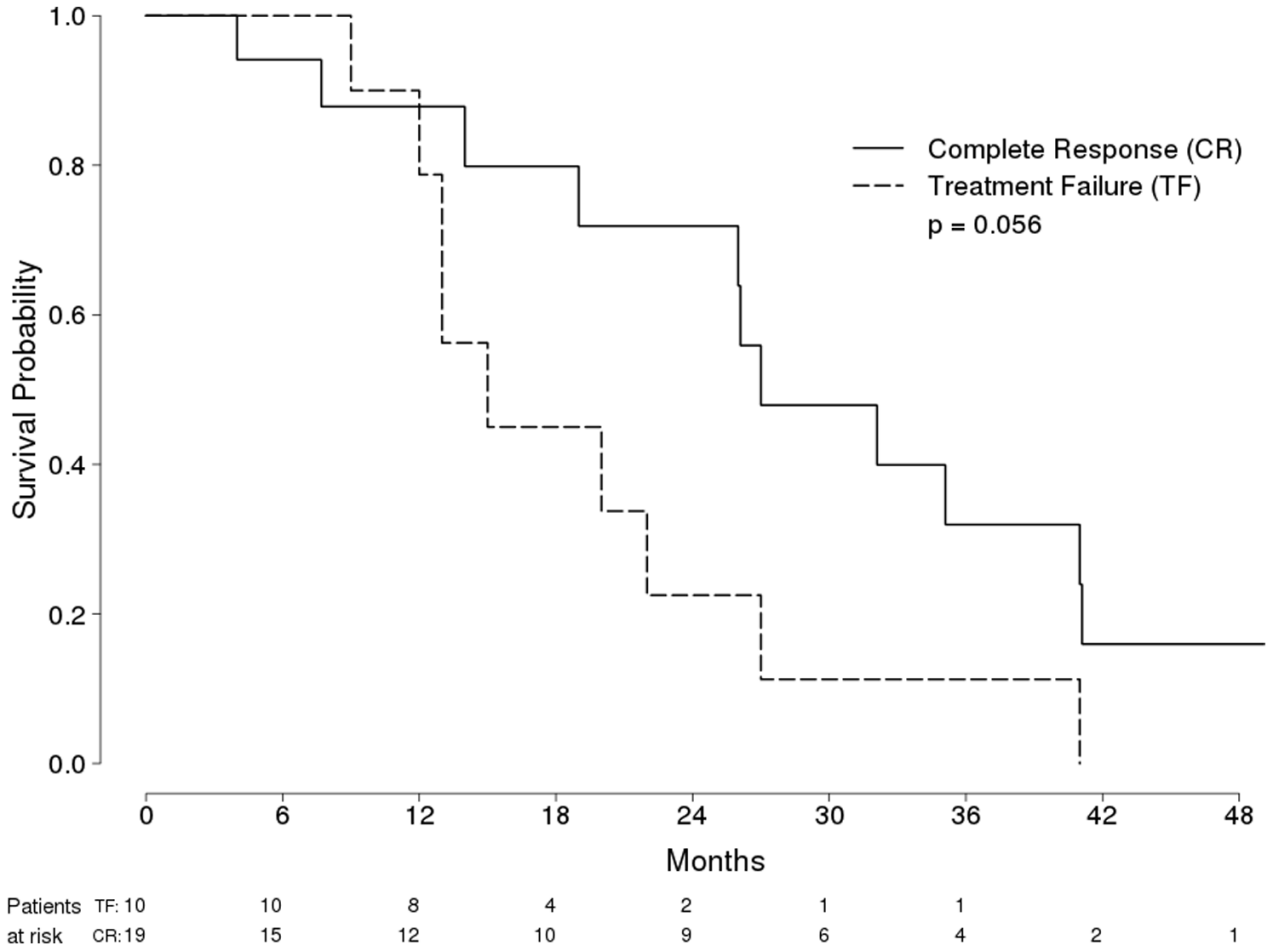
Probability of Overall Survival in 29 Child-Pugh B Patients with HCC Treated with RFA, according to radiological response (Complete response [solid line] vs treatment failure [dashed line]).

Survival was longer among Child-Pugh A class patients (96, 86, 73, 59 and 49% at 12, 24, 36, 48 and 60 months, respectively) than among Child-Pugh B class patients (84, 51, 23, 0.1, and 0% at 12, 24, 36, 48 and 60 months, respectively) (P<0.001 by log-rank test) (Figure 2). Figure 3 and figure 4 show probability of overall survival according to radiological response (Complete response vs treatment failure) in 122 Child-Pugh A Patients and in 29 Child-Pugh B Patients with HCC treated with RFA, respectively.

### Treatment Safety

No patient death was treatment-related. The proportion of major complications after treatment was 4% (Table 2). One patient developed portal vein thrombosis soon after treatment. Other major reported complications were subcapsular hematoma (1 case), intrahepatic abscess (1 case), pleural effusion (1 case), and neoplastic seeding of the needle track (1 cases). The three most frequent minor complications after treatment were pain requiring major analgesia, transient episode of fever and skin burns.

### Analysis of Factors Affecting the Survival of Patients and Complete Response

Cox regression analysis by Cox model showed that complete response after 1 month (HR 0.3; 95% CI: 0.18–0.53; P<0.0001); and albumin levels (HR 0.4; 95% CI: 0.2–0.64; P  = 0.0004) were independent and significant predictors of overall survival (Table 3).

**Table 3:** Predictor of Survival in 151 Patients with HCC in Compensated Cirrhosis Treated with RFA.

Variables (code)	Hazard Ratio	CI 95%	P value
**Complete response after RFA (yes vs no)**	0.3	0.18–0.53	<0.0001
**Albumin-g/dl (continue)**	0.4	0.2–0.64	0.0004

Abbreviations: HR, hazard Ratio; CI, confidence interval; RFA, radio-frequency ablation.

By multivariate analysis, tumor size (≤3 cm vs >3 cm) is the only variable associated with an increased likehood of CR (Odds ratio  = 2.8; 95% CI: 1.2–6.6; P  = 0.01).

## Discussion

HCC secondary to cirrhosis is a complex and heterogeneous disease with wide variations during its clinical course. [9], [10] Surveillance and recal strategy for HCC is now an established practice [2], [11], [12], [13] since early detection of malignant liver nodules is critical to select patients with limited disease, that are potential candidates for curative treatments. Radical treatments (surgical resection, liver transplantation and percutaneous ablation) are usually offered in referral Centres to 30–40% of patients with early-stage HCC, [3] with reported 5-year survival rates of 40–70% for treated patients vs. less than 20% in those left untreated. [3], [9].

In the present prospective study we observed, in a large prospective cohort, that RFA is a safe and effective procedure for the treatment of early stage HCC patients.

The overall survival rate was 41% at 60 months and overall median survival was 47.6 months; complete response was obteined in 78% of patients and we observed a recurrence rate of 66.8%. Our data are consistent with those already published and confirm the importance of RFA in the treatment of patients with HCC at early stage. [4], [14], [15].

Our analysis, specifically designed to identify prognostic factors of survival, shows that, as previously reported, [16], [17], [18] overall survival depends strictly on complete radiological response. This relevant result was already found about ten years ago, by Sala et al. that showed that initial complete response to percutaneous ablation was associated with an improved survival in both Child-Turcotte-Pugh class A and B patients with nonsurgical HCC. In this line, the present paper is a confirmatory contribution on a peculiar aspect that is still valid ten years later its first reporting. [16] So, complete response is a relevant surrogate end-point that should be planned and carefully assessed by multiphasic CT or MR scan in all patients who undergo RFA. [8], [19].

Among the three laboratory parameters of the Child-Pugh score (*i.e.*, albumin, bilirubin, and INR), only the albumin level was found to be significant at multivariate analysis, suggesting that, in addition to cancer-related feature (tumor size), liver function of the underlying cirrhosis must be carefully evaluated before selecting patients for RFA.

This is not novel, but is not a trivial point, because it is well known that cirrhosis underlies HCC in most of the patients and the functional impairment of the underlying liver has a significant impact on prognosis, irrespective of the tumour stage and the effect of tumor therapy.

We also found a correlation of tumor size with an increased likelihood of complete response; this information might help with pretreatment patient stratification in the design of therapeutic trials and in clinical practice therapeutic decisions.

### The Current Study was Affected by Certain Limitations

Lack of histologic data, in supporting radiologic assessment of complete response, suggests that local recurrence could be interpreted as failure to provide adequate local control of HCC, particularly when viable tumor was detected within 6 months from treatment in spite of a radiologic assessment of complete response one month after treatment.

Another weakness of our prognostic model is the lack of data on molecular factors, such as gene expression profiling, which can have some impact on patient’s outcome. [20], [21] However, The predictive ability of our model, including simple and easy clinical and radiological predictors was excellent, underscoring the applicability of our results to new populations and settings, particularly in real clinical practice, where complex and expensive tests are not available.

### What are the Implications of These Results for Current Practice?

In accordance with current guidelines, [2], [3] we confirmed that RFA is a safe and efficacy curative option for the treatment of early stage HCC patients. This is particular relevant whereas the number of patients who undergo liver transplantation is minimal, compared with the total number of patients with HCC, because of the shortage in donors and the stringent transplantation criteria for this indication. [22], [23] Moreover, it is important to remember that the waiting list for OLT is often long and that OLT is not usually indicated in patients >65 years. Therefore, in these two groups of patients, RFA is indicated.

Recently, RCT showed no significant differences in survival rates (overall or disease-free) after RFA or resection; [24] however, because most patients will experience several episodes of recurrence, comparison between resection and RFA is very difficult, especially in terms of their repeatability. In this line, even if different guidelines for the management of HCC provide indications for the use of various treatments as monotherapies, none of these give infication for a multimodal approach (often used in clinical practice) that combines various techniques used, either as first-line therapy or as a rescue (second-line) approach after the failure of a monotherapy. [25], [26] Despite promising results with RFA in monotherapy, the incidence of local and distant recurrence remain challenging. Role of combined locoregional treatment (RFA plus transarterial chemoembolization), especially for nodules >3 cm, should be assessed. [27].

Moreover, given high rate of recurrences after complete response, numerous studies of postoperative adjuvants (tertiary prevention) after resection or ablative therapies have been performed, but the results are not definitive. In this line, an ongoing phase III clinical trial is testing adjuvant sorafenib for prevention of recurrence (STORM trial).

In this study, no procedure-related death was observed and additional major complications occurred (portal vein thrombosis, pleural effusions, intrahepatic abscess, subcapsular hematoma and seeding) in 4% of patients. Complication rate was similar to those usually reported.

In conclusion, the main results of our study are : 1) a complete response after RFA and high baseline albumin level significantly increase survival; 2) the overall survival rate at 60 months is 41%, and remains low because of the high rate of recurrence (67.8%); 3) a tumor size >3 cm significantly affects the response after RFA; 4) RFA appears as a safe and effective treatment for early stage HCC patients.
